# Improving Xylose Fermentation in *Saccharomyces cerevisiae* by Expressing Nuclear-Localized Hexokinase 2

**DOI:** 10.3390/microorganisms8060856

**Published:** 2020-06-05

**Authors:** Liyuan Zheng, Shan Wei, Meiling Wu, Xuehao Zhu, Xiaoming Bao, Jin Hou, Weifeng Liu, Yu Shen

**Affiliations:** 1 State Key Laboratory of Microbial Technology, Institute of Microbial Technology, Shandong University, Qingdao 266237, China; 201820292@mail.sdu.edu.cn (L.Z.); weishan@haut.edu.cn (S.W.); meilingwu@sdu.edu.cn (M.W.); 1911028@tongji.edu.cn (X.Z.); bxm@sdu.edu.cn (X.B.); houjin@sdu.edu.cn (J.H.); weifliu@sdu.edu.cn (W.L.); 2 State Key Laboratory of Biobased Material and Green Papermaking, School of Bioengineering, Qi Lu University of Technology, Jinan 250353, China

**Keywords:** budding yeast, xylose, Hxk2p^S14A^, Mig1p, ethanol

## Abstract

Understanding the relationship between xylose and the metabolic regulatory systems is a prerequisite to enhance xylose utilization in recombinant *S. cerevisiae* strains. Hexokinase 2 (Hxk2p) is an intracellular glucose sensor that localizes to the cytoplasm or the nucleus depending on the carbon source. Hxk2p interacts with Mig1p to regulate gene transcription in the nucleus. Here, we investigated the effect of nucleus-localized Hxk2p and Mig1p on xylose fermentation. The results show that the expression of *HXK2^S14A^*, which encodes a constitutively nucleus-localized Hxk2p, increased the xylose consumption rate, the ethanol production rate, and the ethanol yield of the engineered yeast strain by 23.5%, 78.6% and 42.6%, respectively. The deletion of *MIG1* decreased xylose utilization and eliminated the positive effect of Hxk2p. We then performed RNA-seq and found that the targets of Hxk2p^S14A^ on xylose were mainly genes that encode RNA-binding proteins. This is very different from the known targets of Mig1p and supports the notion that the Hxk2p-Mig1p interaction is abolished in the presence of xylose. These results will improve our understanding of the interrelation between the Snf1p-Mig1p-Hxk2p glucose signaling pathway and xylose utilization in *S. cerevisiae* and suggests that the expression of *HXK2^S14A^* could be a viable strategy to improve xylose utilization.

## 1. Introduction

Lignocellulosic material is considered to be a sustainable source for the production of biofuels and other chemicals. The utilization not only of glucose but also of xylose, which is the second most abundant sugar component in the hydrolysates of lignocellulosic materials, will undoubtedly bring economic benefits [1,2]. *Saccharomyces cerevisiae* is a robust, well studied model organism that is generally recognized as safe and has a strong capacity to metabolize glucose. Furthermore, *S. cerevisiae* is considered a very competitive microbial cell factory for the production of biofuels and chemicals, especially ethanol [1,2,3]. However, *S. cerevisiae* cannot utilize xylose because it does not have the initial metabolic pathway that converts xylose to xylulose, and also because of inefficient xylulose metabolism [1,2,4].

Many previous studies in metabolic and evolutionary engineering have attempted to solve this problem. Mainly, genes from another species of yeast, *Scheffersomyces(Pichia) stipitis*, that encode xylose reductase (XR) and xylitol dehydrogenase (XDH) [1,5,6,7,8] or xylose isomerases (XI) of bacterial or fungal origin [9,10,11,12,13] have been introduced into *S. cerevisiae* to endow it with the capacity to convert xylose to xylulose. Genes encoding xylulokinase (XK) and enzymes in the non-oxidative pentose phosphate pathway (PPP) were overexpressed to improve the efficiency of xylulose metabolism [14]. Furthermore, adaptive evolution was performed in the media using xylose as the sole carbon source to reprogram the regulatory system of *S. cerevisiae* to enhance xylose utilization [13,15,16,17,18,19]. However, despite many successful metabolic and evolutionary engineering strategies, xylose utilization by recombinant *S. cerevisiae* still lags behind its performance on glucose [20] because *S. cerevisiae* lacks a signaling pathway to recognize xylose as a carbon source and to reprogram the cells to convert to a state that promotes xylose utilization.

In several organisms, glucose is not only a nutrient but also functions as a signal molecule in important cellular processes, and it determines the behavior of cells [21]. The main signaling pathways activated by glucose in *S. cerevisiae* include the Snf3p/Rgt2p-Rgt1p pathway that regulates the expression of hexose transporters, the Snf1p-Mig1p pathway that regulates the expression of genes related to non-glucose carbon sources, and the cAMP-protein kinase A (PKA) pathway involved in cellular growth, homeostasis, and the stress response [22]. The activation of these pathways results in the cells being in a state with a high growth rate and high flux in glycolysis and the pentose phosphate pathway but with very low flux in the tricarboxylic acid (TCA) cycle, glyoxylate cycle, gluconeogenesis, and respiration [22]. From a metabolic pathway perspective, this state is also a good state for converting xylose to ethanol. However, many studies have indicated that *S. cerevisiae* does not sense xylose as a fermentable carbon source (such as glucose). Transcriptome studies have suggested that xylose triggers glycolytic metabolism that is not fully activated [20,23]. Only the Snf3p/Rgt2p-Rgt1p pathway shows a slight response to high extracellular levels of xylose, while the other two pathways do not [24,25].

**Figure 1:**
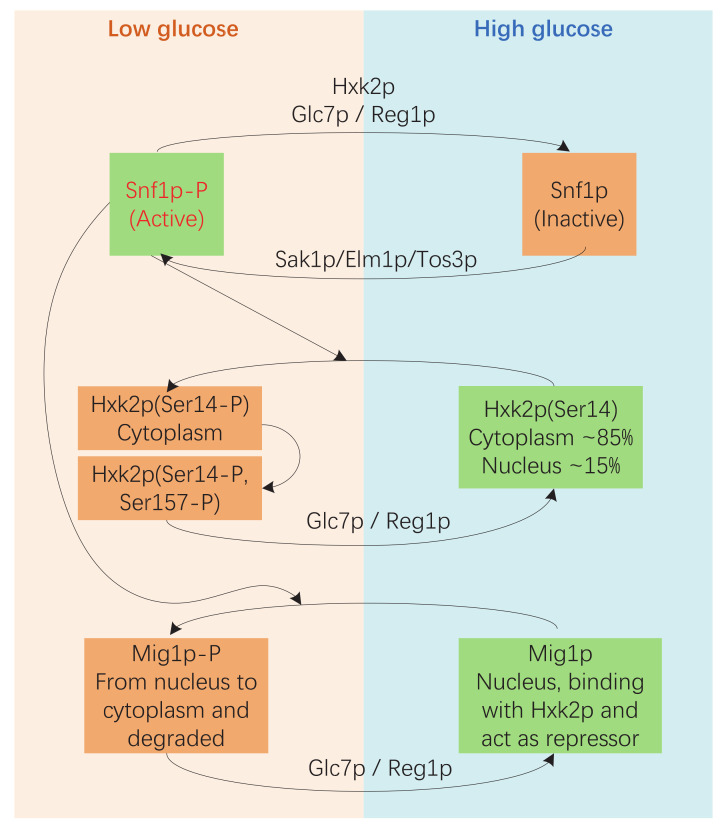
Hxk2p participates in the Snf1p-Mig1p pathway. Under high-glucose conditions, Snf1p is dephosphorylated by the Glc7p-Reg1p protein phosphatase, and Hxk2p is essential for this process. The dephosphorylated form of Snf1p is inactive. Hxk2p and Mig1p are in their dephosphorylated forms and function in glucose repression. In a low glucose environment, Snf1p is phosphorylated by Sak1p/Elm1p/Tos3p. The phosphorylated Snf1p is active and phosphorylates Hxk2p at serine 14, which affects the shuttling of Hxk2p. The active form of Snf1p also phosphorylates Mig1p, and this directly leads Mig1p to move from the nucleus to the cytoplasm. The Mig1p in the cytoplasm is ubiquitinated and degraded by proteases [26,27,28,29].

Corresponding to the regulatory role of glucose, hexokinase acts as an evolutionarily conserved glucose sensor in several organisms, including *S. cerevisiae* [26]. In *S. cerevisiae*, hexokinase 2 (Hxk2p) is not only a glycolytic enzyme in the cytoplasm but also functions as a regulator of gene transcription in the nucleus [21]. Hxk2p is considered to be an intracellular sensor of glucose that participates in the Snf1p-Mig1p pathway [26,27,28,29]. First, Hxk2p is essential in the deactivation of Snf1p by enabling Glc7p/Reg1p to dephosphorylate Snf1p under high glucose conditions. Second, the active form of Snf1p phosphorylates serine 14 of Hxk2p when glucose levels are low. The shuttling back and forth of Hxk2 between the nucleus and the cytoplasm is regulated by phosphorylation and dephosphorylation of serine 14, and the mutant with a non-phosphorylatable alanine at this position (Hxk2p^S14A^) accumulates in the nucleus under low glucose conditions. Third, the nuclear Hxk2p interacts with Mig1p and participates in repressing the expression of genes encoding sugar transporters and the genes needed for utilization of alternative fermentable carbon sources (Figure 1) [21,26]. Moreover, Hxk2p is also involved in the sugar-induced activation of cAMP signaling, and this function is closely associated with the catalytic function of the enzyme. The alteration of Ser158 significantly reduces both the ability of Hxk2p to stimulate the cAMP signal and the hexokinase activity of Hxk2p [29,30,31].

The objectives of the present work were to investigate the effect of nucleus-localized Hxk2p on xylose utilization and ethanol production in *S. cerevisiae*, and to examine the role of Mig1p on this effect. We found that the in situ expression of *HXK2^S14A^*, which encodes a constitutive nucleus-localized Hxk2p, increased the xylose utilization and ethanol accumulation of the engineered yeast strain, while the deletion of *MIG1* decreased xylose utilization. Furthermore, the negative effect caused by deleting *MIG1* eliminated the positive effect of Hxk2p^S14A^ on xylose utilization. We then compared the transcriptomes of the recombinant strain expressing *HXK2^S14A^* and its parent to identify the regulatory targets of Hxk2p^S14A^ on xylose-containing medium. We also compared the targets of *HXK2^S14A^* in cells grown on xylose to the known targets of Mig1p, and the results support the notion that Hxk2p^S14A^ exercises a regulatory function that is independent of Mig1p on xylose. The results presented here will improve our understanding of either the effect of the Snf1p-Mig1p-Hxk2p glucose signaling pathway on xylose utilization in *S. cerevisiae* or the effect of xylose on this signaling pathway. Moreover, our work demonstrates that the expression of Hxk2p^S14A^ is a promising strategy to improve xylose utilization in *S. cerevisiae.*

## 2. Materials and Methods

### 2.1. Yeast Strains and Plasmids

The *S. cerevisiae* strains and plasmids used in the present study are listed in Table 1. The primer sequences are given in Table A1. Strain BSPC039 [5], which overexpresses the *XKS1* and *PPP* genes, was cultured in yeast extract-peptone-dextrose (YPD) medium consisting of 20 g L^−1^ peptone, 10 g L^−1^ yeast extract, and 20 g L^−1^ glucose. Strain BSL01 [25] harboring the plasmid pJX7, which contains an expression cassette for xylose isomerase, was derived from BSPC039 and was used as a control in the present study.

**Figure A1:**
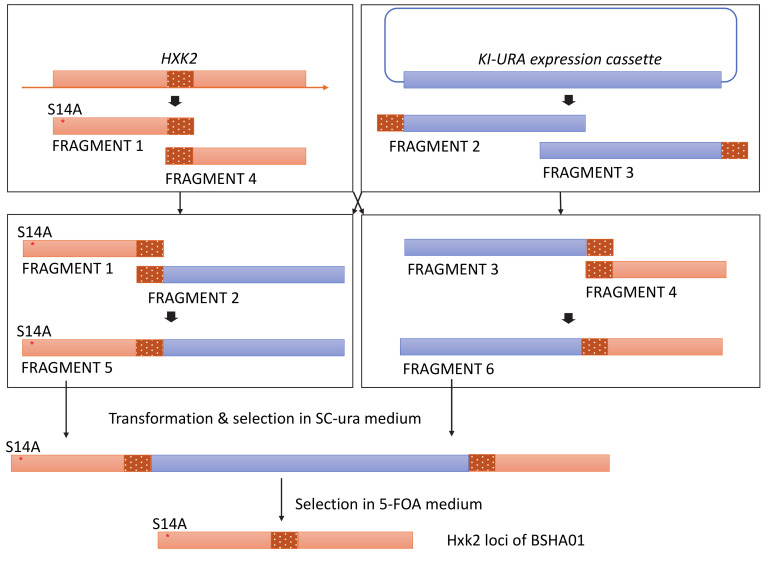
Construction process diagram of the strain BSHA01.

To introduce the mutation S14A in *HXK2*, we amplified and cloned FRAGMENTs 1 and 4 from BSPC039 genomic DNA using the primer pairs Hxk2p-F/DT-hxk2-R(S14A) and DT-hxk2-F(S14A)/Hxk2p-R, respectively, and we amplified and cloned FRAGMENT 2 and 3 from plasmid pUG72 using primer pairs ura-2-F/ura-2-R and ura-1-R/ura-1-F, respectively. We then fused FRAGMENTs 1 and 2 to obtain FRAGMENT 5, fused FRAGMENTs 3 and 4 to obtain FRAGMENT 6, and then transformed BSPC039 with both FRAGMENTs 5 and 6. The transformants were selected on SC-Ura medium (1.7 g L^−1^ YNB-AA/AS, 5 g L^−1^ (NH_4_)_2_SO_4_, 0.77 g L^−1^ CSM-Ura (Sunrise Science Products, USA), and 20 g L^−1^ glucose). The desired transformants (confirmed by sequencing the PCR product of the *Hxk2* locus) were then cultured overnight in YPD medium and selected on SC-Ura agar medium supplemented with an additional 50 mg L^−1^ uracil and 1 g L^−1^ 5-FOA. The clone without the *KI-URA* expression cassette was then transformed with plasmid pJX7 resulting in the strain BSHA01. More details of the construction process are shown in the diagram in Figure A1.

The *MIG1* knockout cassette was amplified from the plasmid YEp-CH [32] using the primers KMig-F and KMig-R. It consists of the homologous recombination arm of *MIG1* and the hygromycin B expression cassette containing the resistance gene *hygB*. We transformed the BSL01 and BSHA01 strains with the *MIG1* knockout cassette. The transformants were selected on SC-Ura medium containing 400 mg L^−1^ hygromycin B to obtain the hygromycin B-resistant strains BSHM01 and BSHM02.

### 2.2. Fermentation

The yeast cells were activated in SC-Ura medium containing 20 g L^−1^ glucose as carbon source. A single colony was inoculated into a 250-mL shake flask containing 30 mL medium and grown at 30 °C with shaking at 200 rpm. After overnight incubation, the cells were transferred to fresh medium with an initial OD_600_ of 0.2 and cultured for another 12–14 h. The activated cells were then collected, washed with sterile water, and inoculated into 30 mL SC-Ura medium containing 20 g L^−1^ xylose as the sole carbon source; the initial biomass was 0.23 g L^−1^ dry cell weight (DCW). Fermentation was performed at 30 °C and 200 rpm in 100-mL shake flasks, and there were three biological replicates for each strain [5].

### 2.3. Analytical Methods

Fermentation samples were collected at specific time intervals. The density of the yeast cells (OD_600_) was determined with a UV-visible spectrophotometer (Eppendorf, Germany). The biomass levels were estimated based on the measured OD_600_-dry weight correlation. One OD_600_ unit corresponded to 0.230 g of DCW L^−1^ for BSPC039 and its derivative strains [5]. The concentrations of xylose and ethanol were determined using a high-performance liquid chromatography (HPLC) system (Shimadzu, Japan) fitted with an Aminex HPX-87H ion exchange column (300 × 7.8 mm) (Bio-Rad, Hercules, CA, USA). H_2_SO_4_ (5 mmol L^−1^) was used as the mobile phase with a flow rate of 0.6 mL min^−1^, and the temperature of the column oven was 45°C [5]. The specific growth rate (μ) was the regression coefficient of the log-linear regression of the OD_600_ versus time during the exponential growth phase [5]. The xylose consumption rate (r_xylose_) and ethanol production rate (r_ethanol_) were the amounts of xylose consumed and ethanol produced per hour per liter, respectively. The t-tests were applied to evaluate the differences between means.

### 2.4. Transcriptome Analysis

Samples for RNA sequencing (RNA-seq) were taken from batch fermentations growing on xylose as the sole carbon source at 14 h. The cells in each sample were collected by centrifugation at 5000 rpm at 4 °C for 5 min and then frozen in liquid nitrogen. Total RNA was extracted using a UNIQ-10 Trizol RNA Purification Kit (Sangon Biotech, Shanghai, China) and then fragmented. Contaminating DNA was removed by digestion with DNase I, and cDNA was synthesized by using short mRNA fragments as templates. Three independent RNA extractions were assayed for each strain. The resulting sample libraries were sequenced using an Illumina HiSeq^TM^ 2000 instrument (BGI, Shenzhen, China). Significant differences were indicated by *p* values ≤ 0.001 and an absolute fold-change threshold of ≥1.5. The information for the Mig1p target genes was obtained from YEASTRACT+ (http://www.yeastract.com/formrankbytf.php) [34].

## 3. Results

### 3.1. Nucleus-Localized Hxk2p^S14A^ Enhances Xylose Fermentation

**Figure 2:**
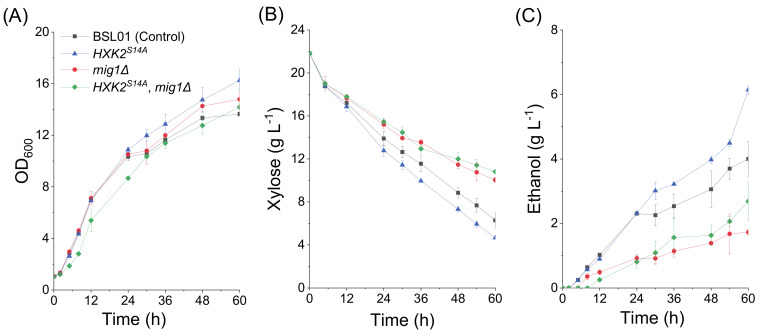
The fermentation characteristics of *S. cerevisiae* recombinant strains cultured in medium containing xylose as the sole carbon source. Fermentation was performed in shake flasks at 200 rpm 30°C with an initial OD_600_ of 1 (biomass ≈ 0.23 g DCW L^−1^). All the data represent the mean value of triplicate tests. Symbols: ■, BSL01 (control); ▲, BSHA01 (BSL01 derivative; *HXK2^S14A^*); ●, BSHM01 (BSL01 derivative; *mig1Δ*); ◆, BSHM02 (BSL01 derivative; *HXK2^S14A^*; *mig1Δ*).

To investigate the role of nucleus-localized Hxk2p in xylose metabolism, the gene *HXK2* in strain BSL01 was replaced by a different allele, *HXK2^S14A^*, that encodes the nucleus-localized mutant Hxk2p ^S14A^ [21], giving strain BSHA01. The xylose fermentation results (Figure 2, Table 2) showed that the xylose consumption rate (r_xylose_), the ethanol production rate (r_ethanol_), and the ethanol yield (Y_ethanol_) of BSHA01 were 0.341 ± 0.027 g L^−1^ h^−1^, 0.118 ± 0.002 g L^−1^ h^−1^, and 0.277 ± 0.032 g g^−1^ xylose, respectively, which are 23.5%, 78.6%, and 42.6% higher than in the control BSL01, respectively. These results suggest that the increase in nucleus-localized Hxk2p has a positive effect on xylose fermentation in recombinant *S. cerevisiae* strains.

### 3.2. Deletion of MIG1 Eliminated the Effect of Nucleus-Localized Hxk2p^S14A^

In high glucose conditions, Mig1p interacts with Hxk2p to form a repressor complex, which represses the expression of genes encoding sugar transporters and the genes needed for the utilization of alternative fermentable carbon sources [21,26]. In order to investigate the effect of Mig1p and the Mig1p-Hxk2p complex on xylose utilization, we deleted the *MIG1* gene in strains BSL01 (control) and BSHA01 (BSL01 derivative carrying the mutant allele *HXK2^S14A^*) to give the strains BSHM01 and BSHM02, respectively. The xylose fermentation results (Figure 2, Table 2) showed that the xylose consumption rate (r_xylose_) of BSHM01 was 0.205 ± 0.010 g L^−1^ h^−1^, which is 25.7% lower than that of BSL01 (0.276 ± 0.034 g L^−1^ h^−1^). Correspondingly, the ethanol production rate (r_ethanol_) of BSHM01 was 0.036 ± 0.004 g L^−1^ h^−1^, which is 45.5% lower than that of BSL01 (0.066 ± 0.003 g L^−1^ h^−1^). The deletion of *MIG1* also decreased the ethanol yield (Y_ethanol_) of the strain BSHM01 by 30.4% (from 0.194 ± 0.015 to 0.135 ± 0.023 g g^−1^ xylose). These results suggest that the Mig1p is not phosphorylated and degraded in the presence of xylose like it is for non-fermentative carbon sources, since the deletion of *MIG1* significantly changed the xylose fermentation characteristics. The strain BSHM02 showed similar xylose fermentation characteristics, but the ethanol yield was higher than in BSHM01. This suggests that the positive effects of Hxk2p^S14A^ on xylose fermentation were eliminated by the deletion of *MIG1*. However, the mutant Hxk2p^S14A^ protein still improved the ethanol production independent of Mig1p.

### 3.3. Transcriptional Profile of the Yeast Strains Expressing Nucleus-Localized Hxk2p^S14A^

To explore how Hxk2p^S14A^ affects xylose metabolism in yeast, we compared the transcriptomic differences between strains BSHA01 and BSL01 cultured in SC-Ura medium with xylose as the sole carbon source. The results showed that 118 genes were up-regulated and 32 genes were down-regulated, respectively, in BSHA01 compared to BSL01 (Table S1).

Analysis of the differentially expressed genes showed that only a small number of the Hxk2p^S14A^-regulated genes are related to carbohydrate metabolism (Table 3). Among them, the only up-regulated gene was *XKS1* that encodes xylulokinase, an enzyme that converts D-xylulose and ATP to xylulose 5-phosphate and ADP. The importance of *XKS1* for xylose fermentation by recombinant *S. cerevisiae* strains has been well established [14]. This could be one reason that Hxk2p^S14A^ enhances xylose fermentation. Furthermore, the down-regulation of *COX2*, *COX3*, and *AI2*, which are involved in respiration, and *GPP1*, which encodes glycerol-3-phosphate phosphatase, might be the reason that Hxk2p^S14A^ increases the ethanol yield of the recombinant strains, since either decreased respiration or decreasing the glycerol production reduced the carbon flux of products other than ethanol.

**Figure 3:**
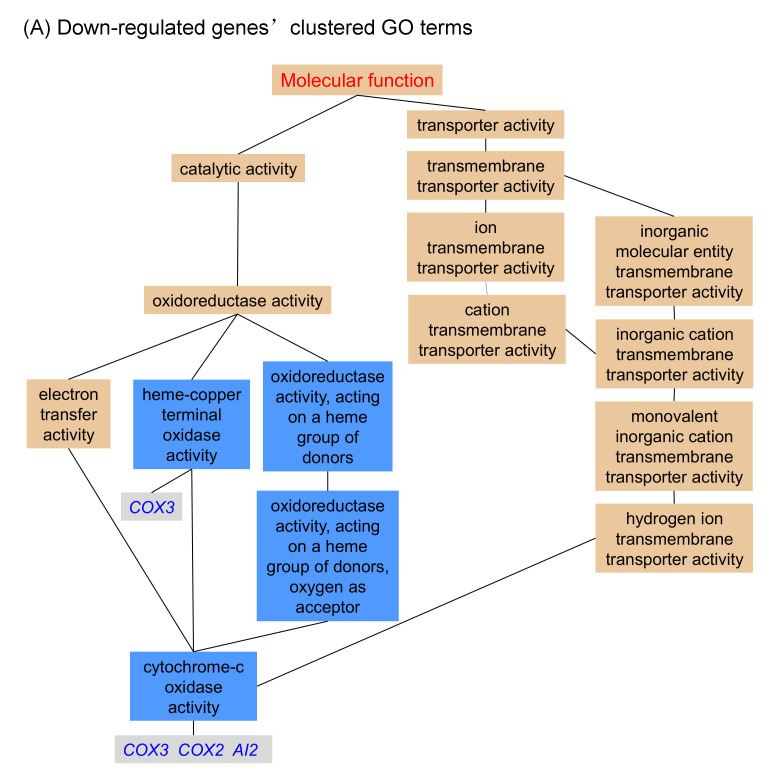

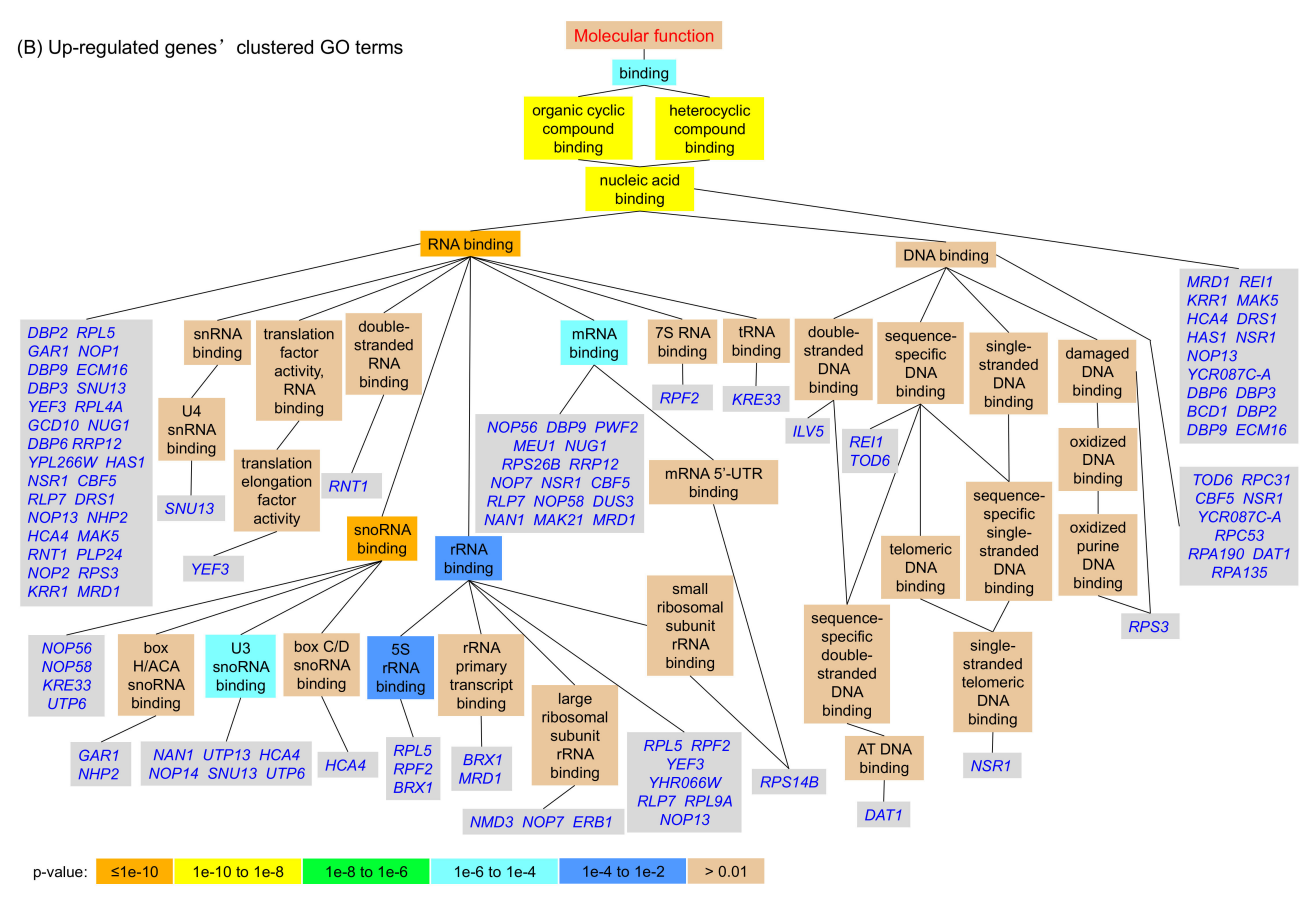
Clustered GO (gene ontology) terms for genes that show significant differential expression in the yeast strain expressing *HXK2^S14A^* compared to its parent strain. (**A**) Down-regulated genes’ clustered GO terms; (**B**) Up-regulated genes’ clustered GO terms.

We then mapped the significant shared GO terms of the genes regulated by Hxk2p^S14A^ using the Gene Ontology Term Finder tool provided by the Saccharomyces Genome Database (http://www.yeastgenome.org). In the ‘molecular function’ GO category (Figure 3), 51.7% of the up-regulated genes (61 of 118) encode nucleic acid binding (GO:0003676) proteins, and most of these (52 of 118 genes, 44.1%) are RNA-binding (GO:0003723) proteins (Figure 3). This result suggested that the transcription and translation processes are more active in yeast strains expressing the nucleus-localized Hxk2p^S14A^ protein compared to the wild type strain. This may be another way to explain the enhanced xylose utilization of the strain BSHA01.

### 3.4. The Regulatory Targets of Hxk2p^S14A^ in Cells Grown on Xylose Are Different than the Known Targets of Mig1p

**Figure 4:**
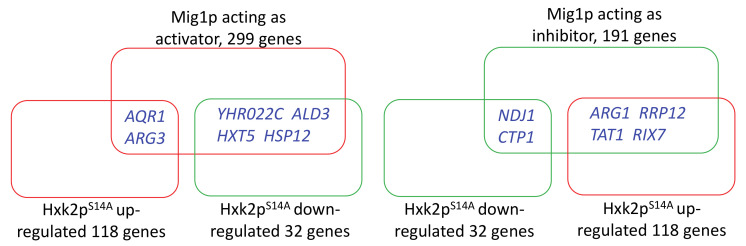
Comparison of the regulatory targets of Mig1p and Hxk2p^S14A^.

Our aim was to analyze the correlation between Mig1p and Hxk2p^S14A^. We compared the regulatory target genes of Hxk2p^S14A^ in cells cultured in xylose, which we obtained from our transcriptome analysis, with the known target genes of Mig1p, which were obtained from YEASTRACT+ (http://www.yeastract.com/formrankbytf.php) [34]. The results (Figure 4) showed that there are only a very few overlaps in the targets of Mig1p and Hxk2p^S14A^. The relative expression of several genes show opposite regulatory profiles by Mig1p and Hxk2p^S14A^. This suggested that, in yeast cells cultured in xylose, the regulatory targets of Hxk2p^S14A^ are totally different than the known targets of Mig1p.

## 4. Discussion

Metabolic and evolution engineering strategies have been performed in *S. cerevisiae*, which is a robust and safe cell factory, to improve its ability to produce ethanol from xylose, which is the second abundant monosaccharide component in lignocellulose hydrolysates [1,2,4]. The state of yeast cells during xylose fermentation, such as the not fully activated glycolytic and pentose phosphate pathway, is very different to that during glucose fermentation. This is due to the response of yeast to xylose being greatly unalike to its response to glucose. Consistent with this, some perturbations to the signaling pathways have shown positive effects on xylose utilization in *S. cerevisiae* [20,25,35,36]. Further metabolic engineering work will require a better understanding of the relationship between xylose and the metabolic regulatory systems in *S. cerevisiae*. Hxk2p is not only a hexokinase but is also an intracellular glucose sensor that plays a significant role in the glucose Snf1p circuit and affects the PKA pathway in *S. cerevisiae*. About 15% of Hxk2p is located in the nucleus under high glucose conditions. It is known that nuclear-localized Hxk2p interacts with Mig1p to form a complex that then regulates the expression of glucose repression genes together. Under low-glucose conditions, Hxk2p moves out of nucleus and does not function as a regulator [29]. In this work, we studied the effects of expressing *HXK2^S14A^*, which encodes the nucleus-localized Hxk2p mutant, and the deletion of *MIG1* on xylose metabolism in recombinant yeast strains. In addition, we also investigated the relationship between the Snf1p-Mig1p-Hxk2p glucose signaling pathway and xylose.

In vitro experiments confirmed that Hxk2p becomes irreversibly inactivated by xylose through an autophosphorylation mechanism in the presence of ATP [37]. Due to the prominent role of Hxk2p in glucose repression signaling, Bergdahl et al. (2013) hypothesized that the inactivation of Hxk2p by xylose leads to a reduced regulatory capability of Hxk2p. They also confirmed that overexpressing the anti-xylose mutation Hxk2p^F159Y^ resulted in an increased rate of xylose uptake and a faster initial growth rate on xylose [29]. Our results show that the expression of Hxk2p^S14A^ enhanced both xylose utilization and ethanol production. This result suggests that the nucleus-localized mutant Hxk2p^S14A^ retains its regulatory function in the presence of xylose, even though it may lose its enzyme activity. The transcriptomic differences between strains expressing Hxk2p^S14A^ and the wild-type Hxk2p also support the notion that Hxk2p^S14A^ possesses a regulatory function. The expression level of *XKS1* in BSHA01 was up-regulated, which may account for the increased xylose utilization, and the expression levels of *COX2*, *COX3*, *AI2*, and *GPP1* were down-regulated, which may explain the increase in ethanol production.

The deletion of *MIG1* decreases xylose utilization, which means that Mig1p is not degraded in the presence of xylose like it is for non-fermentative carbon sources. Otherwise, the *mig1Δ* strain would show no difference compared to the wild-type strain when cultured in xylose. The deletion of *MIG1* eliminated the positive effect on xylose utilization, suggesting that Mig1p is more important than Hxk2p to xylose utilization. Moreover, we found that the regulatory targets of Hxk2p^S14A^ for cells growing on xylose are very different to the known targets of Mig1p. This result supports the hypothesis based on GST pulldown assays that the Hxk2p-Mig1p interaction is abolished by xylose [26]. Considering both of the conclusions of our study ((1) Mig1p is not degraded in xylose, and (2) the Mig1p does not interact with Hxk2p in cells growing on xylose), and the fact that Mig1p is phosphorylated by active Snf1p and then degraded when Mig1p separates from Hxk2p, we further conclude that the activity of Snf1p is not (at least not fully) activated in our strain when cultured in xylose-containing medium so that Mig1p is not phosphorylated by Snf1p and then degraded even it does not interact with the Hxk2p.

## 5. Conclusions

We found that the expression of the nucleus-localized mutant Hxk2p^S14A^ increased xylose utilization and ethanol production in our engineered yeast strain. This may be due to the coordinated up-regulation of *XKS1* and the down-regulation of *COX2*, *COX3*, *AI2*, and *GPP1*. The deletion of *MIG1* decreased xylose utilization and eliminated the positive effect of Hxk2p^S14A^. Most regulatory targets of Hxk2p^S14A^ are RNA-binding proteins in cells grown on xylose, and they are totally different from the known Mig1p targets. Our results reveal the regulatory profile of nucleus-localized Hxk2p^S14A^, which is independent of Mig1p in yeast cells cultured on xylose.

## Figures and Tables

**Table 1:** Plasmids and strains used in this study

Strain and Plasmids	Description	Source
YEp-CH	YEp24 derivative; *GAL1p-Cre-CYC1t*, *TEF1p-hygB-TEF1t*	[32]
pJX7	*2μori*, *URA3*, *TEF1p-RuXI-PGK1t*	[9]
pUG72	*2μori*, *Ampr*, *loxP-KIURA3p-KIURA3-KIURA3t-loxP*	[33]
BSPC039	CEN.PK113-5D derivative; *MATa*; *ura3-52XKS1(-194, -1)::loxP-TEF1p, gre3(-241, +338):: TPI1p-RKI1-RKI1t-PGK1p-TAL1-TAL1t-FBA1p-TKL1-TKL1t-ADH1p-RPE1-RPE1t-loxP*	[5]
BSL01	BSPC039 derivative; pJX7	[25]
BSHA01	BSPC039 derivative; *HXK2^S14A^*, pJX7	This work
BSHM01	BSL01 derivative; *mig1Δ*, pJX7	This work
BSHM02	BSHA01 derivative; *mig1Δ*; *HXK2^S14A^*, pJX7	This work

**Table 2:** Xylose fermentation characteristics of the parental and engineered yeast strains.

Strains (Genotype)	μ (h^−1^)	r_xylose_ (g L^−1^ h^−1^)	r_ethanol_ (g L^−1^ h^−1^)	Y_ethanol_ (g g^−1^ xylose)
BSL01 (Wild type)	0.153 ± 0.003	0.276 ± 0.034	0.066 ± 0.003	0.194 ± 0.015
BSHA01 *(HXK2^S14A^*)	0.163 ± 0.008	0.341 ± 0.027*	0.118 ± 0.002*	0.277 ± 0.032*
BSHM01 (*mig1Δ*)	0.155 ± 0.010	0.205 ± 0.010*	0.036 ± 0.004*	0.135 ± 0.023*
BSHM02 (*HXK2^S14A^*; *mig1Δ*)	0.145 ± 0.047	0.200 ± 0.024*	0.050 ± 0.014*	0.169 ± 0.035

μ, specific growth rate; r_xylose_, volumetric xylose consumption rate; r_ethanol_, volumetric ethanol production rate; Y_ethanol_, ethanol yield. The values are given as the averages and standard deviations of three independent measurements of the fermentation results for cultures grown in 2% xylose at 30^o^C with shaking (200 rpm). * *p*-value < 0.05.

**Table 3:** Hxk2p ^S14A^-regulated genes that were annotated to GO terms related to carbohydrate metabolism in the ‘Biological Process’ GO category.

GO Term (GO ID)	Up-Regulated Genes	Down-Regulated Genes
generation of precursor metabolites and energy (GO:0006091)	-	*ADH4*, *AI2*, *COX2*, *TKL2*
cellular respiration (GO:0045333)	-	*AI2*, *COX2*, *COX3*
carbohydrate metabolic process (GO:0005975)	*XKS1*	*GPP1*
carbohydrate transport (GO:0008643)	-	*HXT5*
monocarboxylic acid metabolic process (GO:0032787)	-	*ALD3*

## Supplementary Materials

The following are available online at https://www.mdpi.com/2076-2607/8/6/856/s1, Table S1: The significantly diff-expressed genes of strain-expressing *HXK2^S14A^* (HA) vs. the parent strain (WT).

## Appendix A

**Table A1:** Oligonucleotide primers used in this work.

Primers	Sequence5′→3′
Hxk2p-F	CTCCAGAGCTCCACATTGGTG
DT-hxk2-R(S14A)	GGCACATCGGCCATGGCACCCTTTCTGGCTTG
DT-hxk2-F(S14A)	CAAGCCAGAAAGGGTGCCATGGCCGATGTGCC
HXK2-R	ATCGTCACGAATAAATCCCGTG
ura-1-F	CCATCTGCTCCAATGGCCATCAACTGTGAATACGG TTCCGTGATTCTGGGTAGAAGATCGGTC
ura-1-R	GAACCCTTGGAAGACAATTCAGC
ura-2-F ura-2-R	GAAACGTTGGGTCCCATACATTTG GAAAGAAAAGGTGAAACCCAATGGAATTGGCTCA GAGATACCCGAAGTTATTAGGGTCTAGAGATCCC
KMig-F	CAGCAGAAAAGCGCAATTGCGACACTAGCAGTGTA ACTCGTAGGTCTAGAGATCTGTTTAGCTTG
KMig-R	GAAGGTGGAGGCTTCACCGAGACGGGAATCTTAGC CATCGATTAAGGGTTCTCGAGAGCTC
